# Culturally Sensitive Perinatal Mental Health Care: Experiences of Women From Minority Ethnic Groups

**DOI:** 10.1111/hex.14160

**Published:** 2024-08-01

**Authors:** Angelene Gardner, Sheri Oduola, Bonnie Teague

**Affiliations:** ^1^ Department of Clinical Psychology and Psychological Therapies, Norwich Medical School University of East Anglia Norwich UK; ^2^ School of Health Sciences University of East Anglia Norwich UK; ^3^ Research and Development Norfolk and Suffolk NHS Foundation Trust Norwich UK

**Keywords:** cultural sensitivity, culture, experiences, mental health services, minority, perinatal mental health, qualitative study

## Abstract

**Background:**

Current research has identified how ethnic minority women experience poorer health outcomes during the perinatal period. In the United Kingdom, specialist perinatal mental health services provide mental health treatment for women throughout the perinatal period. Service users have previously highlighted that perinatal services are hard to access and lack cultural sensitivity, whereas healthcare professionals have described limited opportunities and resources for developing cultural competency.

**Objectives:**

We explored the experiences of ethnic minority women with National Health Service (NHS) specialist perinatal teams and identified what culturally sensitive perinatal mental health care means to this group.

**Design:**

Individual semi‐structured interviews were conducted, and an interpretative phenomenological analysis framework was used to analyse the interview transcripts.

**Setting and Participants:**

Participants were recruited from NHS specialist perinatal teams and online via social media.

**Results:**

Six women were interviewed. Four group experiential themes central to the experiences of participants emerged: (1) strengthening community networks and peer support; (2) valuing cultural curiosity; (3) making sense of how culture, ethnicity, race and racism impact mental health; and (4) tailoring interventions to ethnic minority women and their families.

**Discussion and Conclusions:**

The findings capture how ethnic minority women experience specialist perinatal teams and offer insights into practising culturally sensitive care. Perinatal mental health professionals can support ethnic minority women by strengthening their access to community resources and peer support; being curious about their culture; helping them to make sense of how culture, ethnicity, race and mental health interact; and applying cultural and practical adaptations to interventions.

**Patient or Public Contribution:**

A Lived Experience Advisory Group (LEAG) of women from ethnic minority groups contributed to the design and conduct of this study. The LEAG had lived experience of perinatal mental health conditions and accessing specialist perinatal teams. The LEAG chose to co‐produce specific aspects of the research they felt fit with their skills and available time throughout five group sessions. These aspects included developing the interview topic guide, a structure for debriefing participants and advising on the social media recruitment strategy.

## 1 Introduction

The perinatal period spans from pregnancy to 12 months after childbirth, during which approximately one in five women experience perinatal mental health difficulties [1]. Preliminary research suggests that ethnic minority women are significantly more likely to experience perinatal mental health difficulties compared to White British women [2, 3] (National Childbirth Trust 2015), and ethnic minority women are twice as likely to have unidentified mental health difficulties [4].

Furthermore, the Mothers and Babies: Reducing Risk through Audits and Confidential Enquiries across the United Kingdom (MBRRACE UK) report found that women of Asian and mixed ethnicity backgrounds are twice as likely to die during pregnancy compared to White women, whereas mortality rates in Black women are four times higher than White women [5]. The National Health Service (NHS) Long‐Term Plan [6] has emphasised the need to reduce these health inequalities and increase continuity of care during the perinatal period. Following this, the Core20PLUS5, which focuses on the most deprived 20% of the UK population with five priority areas for improving healthcare inequalities, has identified ensuring continuity of care for Black, Asian and mixed ethnicity women in maternity services as a priority [7].

Perinatal mental health services are specialised services within secondary mental health care that provide treatment for women who are pregnant or have a child up to 12 months old and are experiencing serious mental health difficulties [8]. Evidence suggests that further research is required to improve culturally sensitive care in perinatal and maternity services [9, 10]. Culturally sensitive care is the conscious understanding and appreciation of values, norms and beliefs inherent in cultural, ethnic or racial groups; it entails openness and willingness to adapt one's behaviour and perspectives [11]. Culturally sensitive care in perinatal services is crucial because culture influences how perinatal mental health difficulties are presented and how individuals make sense of their difficulties and their relationship with seeking help [12].

From the perspectives of healthcare professionals in perinatal services, a lack of cultural sensitivity and culturally adapted interventions increases care inequalities between different groups [13]. Claeys et al. [10] interviewed nurses, midwives and healthcare students about their perceptions of culturally sensitive care. They reported that professionals shared difficulties in adopting culturally informed practice, a tendency to compare patients from different cultural backgrounds against their cultural frame of reference and a lack of readily available knowledge on cultural competency [10]. Edge [9] explored the views of NHS perinatal mental health professionals on service provision for ethnic minority women and the extent to which they observed services meeting their needs. Similar to Claeys et al. [10], professionals reported a lack of culturally appropriate resources and treatments, and some professionals shared a reliance on signposting women to external culturally specific services to meet their needs [9].

Furthermore, a systematic review by Watson et al. [14] synthesised findings from 15 UK studies investigating ethnic minority women's experiences with maternity and perinatal services. Barriers to accessing services included lack of awareness, language barriers and fear of judgement regarding mental health status [14]. After accessing services, women reported encountering cultural insensitivity and interpreter unavailability and described staff as ethnically homogenous [14]. Two recent qualitative studies explored ethnic minority women's experiences with UK specialist perinatal teams [15, 16]. Pilav et al. [15] recruited women from Black, Asian, Arab, mixed ethnicity and White Other backgrounds from a perinatal service in South London. The findings of Pilav et al. [15] highlighted barriers to support across individual and societal levels. Pilav et al. [15] suggested that the intersectionality between cultural and societal expectations of motherhood and mental health difficulties increased psychological distress. Conneely et al. [16] explored the experiences of Black and South Asian women with predominantly Birmingham and London–based perinatal services and identified several factors that impacted accessing help, including self‐identity, social expectations, attributions of distress and perceptions of services as hidden. Conneely et al. [16] identified that professional curiosity, kindness and flexibility were beneficial.

Despite known inequalities in perinatal services, there has been no research that asks ethnic minority women for their perspectives on how they would define culturally sensitive care in the context of perinatal mental health services. Furthermore, this study was conducted in the East of England, an underrepresented area in research where ethnic minorities account for a much lower proportion of the population compared to the recruitment settings included in the current literature. The following research questions were addressed: What are ethnic minority women's experiences of perinatal mental health services in the East of England? How do ethnic minority women conceptualise culturally sensitive care?

## 2 Methods

This study followed the Consolidated Criteria for Reporting Qualitative Research (COREQ; see Supporting Information) [17], a 32‐item checklist to improve the quality of reporting in qualitative research.

### 2.1 Design

This study implemented a qualitative semi‐structured interview design, and an interpretative phenomenological analysis (IPA) was chosen for data analysis [18, 19]. IPA focuses on a detailed exploration of participants' lived experiences and meaning‐making processes within their personal, social and cultural contexts [20]. IPA is interested in first understanding the nuances and complexities of individual participants before identifying patterns of convergence and divergence across cases [21]. IPA's strengths of collecting first‐hand accounts of personal experiences and meaning‐making [20, 22] were most suitable for our research aims which include understanding experiences of perinatal services and conceptualisations of culturally sensitive care.

### 2.2 Setting

All participants in the current study have accessed NHS perinatal mental health teams in the East of England. In this region, ethnic minority groups constitute only 21.5% of the population, and non‐White ethnic minorities account for only 13.5%, in contrast to London, where ethnic minorities account for 63.2% of the population [23].

### 2.3 Participants

The following inclusion criteria were applied: Women accepted onto the caseload of community perinatal teams and/or mother and baby units in the East of England over the past 2 years and all women who identify as being from minority ethnic groups and/or mixed ethnicity groups, over the age of 18 and English speaking to a level they can follow and engage in research activities.

A convenience sampling strategy was used to select participants from six NHS perinatal teams across the East of England and to advertise the study poster on social media channels, including Facebook, Twitter, Instagram and charity websites. For NHS recruitment, the primary researcher (A.G.), a trainee clinical psychologist, attended perinatal services' multidisciplinary team meetings to advertise the research study and liaised with clinicians throughout the recruitment period from 1 April 2023 to 1 October 2023. Perinatal team clinicians provided consent to contact forms to service users who met the predetermined inclusion criteria. Interested service users entered their details into the form and provided their signatures to consent to AG making contact; following this, their clinicians emailed the consent forms to A.G. Participants recruited through social media emailed AG directly.

A.G. conducted pre‐engagement telephone calls to discuss the context of the research. A.G. shared her ‘insider’ positionality in terms of being a mother from a mixed ethnic background with lived experience to demystify herself as the researcher, build rapport and help participants feel empowered to share their stories [24, 25]. Following this, participant information sheets were shared and participants were allowed to ask further questions before entering into the study.

The study recruited six participants and no participants refused to participate or dropped out of the study. Pseudonyms have been used throughout this study to preserve anonymity (Table 1). Similar sample sizes have been employed in recent studies using an IPA approach to explore the lived experiences of ethnic minorities [26, 27, 28]. Smaller sample sizes allow for a more in‐depth analysis to accommodate the rigorous methods involved in IPA [20].

**Table 1:** Participant characteristics.

Participant alias	Ethnicity	Cultural background	Age	Mental health difficulty	Time under perinatal service	English as first language
Farah	Mixed ethnicity background: Moroccan, Irish and English	British and Islamic family background	34	Depression and anxiety	12 months	Yes
Inaya	British Pakistani	Muslim and Punjabi	31	Depression	12 months	Yes
Ishani	Indian	Malayan and catholic	37	Birth trauma	5 months	No—Malayalam
Alesha	Mixed ethnicity background: Jamaican, Irish and English	Agnostic and spiritual, raised Catholic	30	Antenatal depression and anxiety, birth trauma	5 months	Yes
Nasrin	Bangladeshi	Hindu	30	Depression	6 months	No—Bangla
Zoya	British Pakistani	Islam	36	Anxiety and depression	11 months	No—Punjabi

### 2.4 Data Collection

The interview topic guide (Table 2) focused on pathways to care, experiences of care, cultural and ethnic identity and strategies for improvement. Before facilitating interviews with participants, A.G. received interview skills training and practised the interviews with the Lived Experience Advisory Group for the study. Due to their preference, four participants were interviewed online and two were interviewed in person, at their homes. Only participants, their babies and A.G. were present during the interviews. No repeat interviews were carried out. The interviews and debriefing duration ranged from 60 to 90 min and took place over 5 months. Interviews were audio recorded, transcribed verbatim and anonymised.

**Table 2:** Interview topic guide.

Topics	Examples of interview questions
Pathways to care	Please tell me about your journey into the community perinatal team/mother and baby unit.
Experiences of care	Overall, how was your experience of X team/teams? To what extent do you feel you were involved in decisions about your care?
Cultural and ethnic identity	How important do you feel your ethnic background is to your health? To what extent do you feel your ethnicity or culture might have influenced the care you received? And why? What does culturally sensitive care mean to you?
Strategies for improvement	What were your needs from the service and were they met?

### 2.5 Analysis

Following the IPA approach, A.G. analysed each transcript individually before conducting a cross‐case analysis. Each audio recording was listened to and transcripts were read several times; following this, descriptive, linguistic and conceptual exploratory notes were made [20]. As recommended in IPA, qualitative analysis software was not used, and all transcripts were printed and annotated by hand [29]. Exploratory notes were formulated into experiential statements, which were grouped to make personal experiential themes. These were compiled into a table alongside relevant quotes to ensure that the narrative was grounded in the data. Once each case was completed, all participants were emailed their tables of personal experiential themes and statements for their feedback. None of the participants requested changes to be made.

Following this, cross‐case analysis began to create group experiential themes from the personal experiential themes and statements. Initial patterns of group experiential themes were formulated as a research team, and the primary researcher finalised the table of group experiential themes. A reflective diary was used throughout the research process, and any questions about the data, preconceptions and key insights were reflected on as a research team.

## 3 Results

From the analysis of the six interview transcripts, four group experiential themes central to the experiences of participants emerged: (1) strengthening community networks and peer support; (2) valuing cultural curiosity; (3) making sense of how culture, ethnicity, race and racism impact mental health; and (4) tailoring interventions to ethnic minority women and their families. The presence of these themes within each individual's account is highlighted in Table 3. A detailed description of each theme accompanied by participants' quotes follows.

**Table 3:** Table of group experiential themes and their relationship to participants.

Theme	Farah	Inaya	Ishani	Alesha	Nasrin	Zoya
Strengthening community networks and peer support	✔	✔	✔		✔	✔
Valuing cultural curiosity	✔			✔	✔	✔
Making sense of how culture, ethnicity, race and racism impact mental health	✔	✔	✔	✔		
Tailoring interventions to ethnic minority women and their families	✔	✔	✔	✔	✔	✔

### 3.1 Group Experiential Theme 1: Strengthening Community Networks and Peer Support

Women described the beneficial role of perinatal teams in helping them strengthen their community networks and providing them with support from peers with lived experience. This was seen as crucial for those experiencing acculturative stress and isolation.

Farah shared the importance of perinatal teams helping service users strengthen their social networks. She reflected on this being consequential because she perceived parenting in England to be increasingly self‐reliant, as opposed to the interdependent approach observed more commonly in non‐Western cultures. Similarly, Ishani shared the challenges of living with reduced support in England:

It is very typical in our Western culture, the women are quite isolated and this nuclear family is really praised and like doing it on your own and people don't live near their family. So I think a service that can recognise that is the culture we have today, whether we like it or not and try and help tap people as quickly as possible into a community […] You know, if I just meet women once a week … those negative, lonely, isolating thoughts go away …

(Farah)

… back home, we have a lot of support and here we don't. I think a lot of women do suffer here, you know, in silence, they don't talk about their feelings. They're meant to get on with it basically.

(Ishani)

Farah and Ishani articulated a cognisance of the challenges inherent in parenting without the help of extended family and established social networks. Interestingly, neither Inaya, Alesha nor Nasrin mentioned receiving support from their partners or wider family. It appears that strengthening social networks is imperative due to the lack of family support experienced by this group. Farah and Ishani suggested that managing parenting without practical, emotional or social support from others is unsustainable. Their accounts implied that they both had experienced isolation during the perinatal period and distancing themselves by sharing their perceptions of other women experiencing this isolation alludes to their feelings of vulnerability.

In contrast, participants who illustrated their perinatal team's involvement in strengthening community networks and peer support conveyed a heightened sense of connectedness, reduced isolation and confidence. Nasrin was given a peer support worker to help her develop confidence in going outside. Inaya and Zoya shared how their perinatal teams engaged in multiagency working to help them expand their professional and peer support networks within their communities:

I have a Peer Support Worker who helped me get out and afterwards I feel more confident.

(Nasrin)

There were quite a few projects with Muslim mums *[…]* that kind of stuff for like Mum's spirituality.

(Inaya)

And then *[care coordinator's name]* told me about Mind *[mental health charity] […]* She *[Mind practitioner]* was amazing. She was seeing me every Monday … Talked to me, asked how I'm feeling. Whoever I met in this journey, they were amazing. So she was amazing. But actually she said she went through the same. She knows all the experience. She went through all this as well. So in our culture, we don't really think about this thing, you know about mental health, that in our culture it doesn't exist … 

(Zoya)

It was apparent that having regular sessions through a charitable organisation providing mental health support helped Zoya feel cared for. Her account suggests that being seen by a professional with lived experience normalised mental health difficulties in the context of her experiences of cultural stigma towards mental health and helped build her sense of connection with others. Furthermore, Zoya's perinatal team helped her identify existing members of her support network. Zoya shared the value of relying on a close support network to find her comfort and to help manage her family life and routine at a time when her depression was most severe. Learning to accept help from others was a valuable learning experience for Zoya:

My neighbours and my friends was amazing. Picking up the kids and dropping off the kids. Honestly, just take it, whoever wants to offer you anything. Just take it because I was like no, no, no, I'm fine. I don't need any help. I'm okay. But now I just take anybody's help.

(Zoya)

Participants' responses revealed the significance of viewing the mother and the baby as embedded in their broader social and community contexts. Participants whose perinatal teams were actively helping them strengthen their existing social and peer support networks and create new ones appeared to have a more beneficial impact than those who were less connected.

### 3.2 Group Experiential Theme 2: Valuing Cultural Curiosity

Women expressed the value of professionals being culturally curious and asking them questions about their cultural background. There were apparent differences in the extent to which participants experienced this curiosity.

Nasrin and Zoya described how culture was discussed in detail throughout their therapy sessions and home visits from staff. Zoya described how staff were culturally attuned and asked them questions about key religious events:

I enjoy someone who wants to know about my culture … Curiosity makes me happy. Yeah, because I'm working with someone who has an interest in me.

(Nasrin)

They asked me what I'm doing for Eid and ‘did you make clothes for the kids?’

(Zoya)

It was clear that for Nasrin and Zoya, asking about culture and cultural festivities was important for building relationships with professionals. Conversely, Farah and Alesha experienced professionals as neither open nor competent to talk about culture. Farah described providing data on her ethnic background when entering the perinatal service but was later disappointed when her ethnicity was not explored further:

… first time you're accessing services, not just asking about your ethnicity for a tick box exercise. […] But actually, yeah, exploring that, spending a few minutes trying to understand, you know, I present as mixed‐race, somebody else might present as mixed‐race, but our upbringing could be quite different.

(Farah)

Alesha shared how she experienced perinatal team staff as visibly uncomfortable when culture and race became present and expressed that she had wanted this to be explored:

I would just like someone to actually acknowledge it before I do sometimes. Because then I can see people getting awkward because they don't know how to talk. And it's like the elephant in the room sort of thing. So, it would just be nice to have people say, you know, oh, ‘how have you found the service as a mixed‐race woman or like, you know, has anything ever come up for you?’ It'd just be nice for people to check in because it's an important part of your identity.

(Alesha)

It is clear from participants' accounts that their culture, ethnicity and race are integral parts of their identity. Farah's and Alesha's experiences suggest that professionals not exploring culture, ethnicity and race may be perceived as rejecting their identities and exacerbating a feeling of discomfort, prohibiting good relationships from forming with staff. Farah and Alesha suggest that asking about ethnicity beyond data collection and professionals conveying an interest in understanding how diverse women perceive services can help improve experiences for ethnic minority women. Despite differences in the extent to which women felt they received cultural curiosity from professionals, they all felt this was important for their care.

### 3.3 Group Experiential Theme 3: Making Sense of How Culture, Ethnicity, Race and Racism Impact Mental Health

Participants similarly expressed the importance of being able to explore how their culture and mental health difficulties influence one another. Similar to the prior theme, there were differences in the extent to which participants had been supported by specialist perinatal staff to explore and formulate the impact of culture, ethnicity, race and racism on their mental health.

Inaya, Ishani and Farah each described the influence of their culture and upbringing on their mental health. Inaya reflected on cultural stigma towards mental health difficulties within her culture and how these judgements negatively affected her mental health in the past:

… get labelled as crazy. Not, not on purpose, but let's say if someone got depression, so they must be crazy. But they're all words that don't help.

(Inaya)

Ishani also described when cultural norms impacted her mental health, but with a focus on childhood trauma:

If you are not listened to as a child, for example, quite normal there *(home country)*. And you come here, and you look at it and say, ‘you know, that's just a child and you didn't have to go through all that’.

(Ishani)

Ishani shared that she was supported in discussing her cultural upbringing during formulation sessions with a psychologist from the perinatal team. She noticed the benefit of her psychologist being from outside of her culture. Ishani believed this difference in cultural outlook on childrearing practices meant that the psychologist was able to identify that Ishani suffered child abuse, and it was evident that Ishani felt validated by this. Ishani suggested that someone within her own culture may not have recognised this as abuse as she inferred that child maltreatment is normalised in her culture. Being supported to discuss her cultural upbringing and acknowledge the childhood abuse she experienced was interpreted as having supported Ishani to develop compassion towards her difficulties. Farah also discussed the impact of her cultural upbringing but reflected on how this influenced her belief system:

… my brains been wired from when I was younger and maybe you know, again bringing up my upbringing and certain kinds of beliefs and, or, certain limiting beliefs. For me, all of that is so part of culture. So, part of like why, you know … Why I think certain things, why you're so kind of harsh on yourself, why you're so negative … 

(Farah)

By noticing the influences of their cultural upbringing on their well‐being, it was interpreted that Farah and Ishani perceived themselves as having an opportunity to make sense of their difficulties and identify their need for self‐compassion.

Making sense of the influence of race and racism was identified as particularly pertinent for Alesha. Alesha expressed the importance of professionals acknowledging critical events in the media about perinatal outcomes and race. Alesha referred to the MBRRACE report's finding that mortality rates for Black women are four times higher than for White women [5]. She reflected on the need for professionals to ask her about how these events have impacted her:

We know that women from minorities have a lower level of care […] There's research into it, like we know it's a thing. And we know that a lot of times people are ignored in times of pain. And so it's even just like oh, ‘how do you feel about this stuff that's come out *[MBRRACE‐UK findings [* 5 *]]*?’ Because I was terrified when I was giving birth to *[baby's name]*, I was straightening my hair all the time, thinking I want to be more racially ambiguous, which is horrible to me … I think it's important for women to talk about our birth experience and experiences of racism.

(Alesha)

Alesha highlighted how she felt the MBRRACE UK statistics were close to reflecting her childbirth experience. Alesha's account suggests that perinatal teams being aware of current affairs and systemic racism is meaningful. Alesha and Farah shared that perinatal staff had not asked about their race, ethnicity and culture. During the interviews, Alesha and Farah were seen to be overtly engaging in active meaning‐making. At the end of the interview, they shared that professionals had not discussed these topics with them and reflected on how the research interview acted as a sense‐making intervention.

Exploring experiences of culture, ethnicity, race and racism were valued by the women included in this study. Women whose healthcare professionals had facilitated these conversations expressed the following: being supported to make sense of how their upbringing impacts them today, having help to identify childhood abuse, and being able to acknowledge cultural stigma and distance themselves from it.

### 3.4 Group Experiential Theme 4: Tailoring Interventions to Ethnic Minority Women and Their Families

Critically reflective practice was considered necessary when working with ethnic minority women. All women suggested various cultural and practical considerations for working with women from ethnic minority groups. However, there were differences in what women appraised as most important for their care.

Farah shared her experiences of cognitive behavioural therapy and reflected on how integrating her cultural context into therapy would have been beneficial. She conveyed her perspective that evidence‐based perinatal mental health treatments often do not fit women from ethnic minority backgrounds. Farah recommended clinicians apply problem‐solving and critical reflection to adapt treatment for ethnic minority women:

I think a lot of these kind of structures and frameworks are probably created traditionally by like white British males. So, when you look at that kind of systemically, and then somebody isn't responding to a treatment, you've gotta kind of look outside the box a bit.

(Farah)

Inaya, Zoya, Ishani and Nasrin all placed value on speaking with clinicians of the same cultural and linguistic background. Zoya described the ease of communicating in the same language with her psychiatrists:

Yeah, you know, this language barrier does come up, I try to speak very well, but sometimes you know, it helped to tell them in my own language.

(Zoya)

Ishani shared that coming from the same linguistic and cultural background as her therapist would enable her to converse intuitively and express her thoughts and feelings with ease:

When you're trying to find words in English, which is a common language for you, it's kind of ermm difficult to express what you really feel […] You're finding words, it's not from the heart, it's from the brain.

(Ishani)

When no clinicians spoke the same language, Inaya described her husband benefitting from an interpreter when attending a group for dads:

He needed a translator for meetings and a few sessions with other dads.

(Inaya)

Although Nasrin also felt she may have benefitted from a therapist who matched her cultural and linguistic background, she described a strong therapeutic relationship with her therapist. She thought an English‐speaking therapist was acceptable, provided they made adaptations. Nasrin shared that she spent many assessment sessions talking and explaining her culture to her therapist. She thought that the number of intervention sessions was limited due to this extended cultural assessment. She acknowledged that therapy within the NHS is often time‐limited but suggested an increased number of sessions in these instances for equitable care:

… I think extended sessions for ethnic minority is also helpful. Yeah. Because our culture is different, so they needed more time to understand me.

(Nasrin)

Alesha was the only participant to recommend a new intervention for ethnic minority mothers under the care of the perinatal team to discuss complex cultural narratives and the influence of race on their experiences:

It would be good to have a group specifically for minority women, it might involve talking about different experiences of motherhood, family and how our race has impacted us.

(Alesha)

The data suggest that there is no ‘one size fits’ approach to tailoring care for ethnic minority women from different cultural backgrounds; however, participants' responses suggest that practical and flexible adaptations are needed to suit the needs of minority groups.

## 4 Discussion

The study investigated the experiences of ethnic minority women with specialist perinatal teams and how they conceptualise culturally sensitive care. To our knowledge, this is the first study that asks ethnic minority women what culturally sensitive care means to them. Four group experiential themes were developed from the six participants' accounts: (1) strengthening community networks and peer support; (2) valuing cultural curiosity; (3) making sense of how culture, ethnicity, race and racism impact mental health; and (4) tailoring interventions to ethnic minority women and their families.

Strengthening community networks and peer support was conceptualised as integral for participants' recovery. Research suggests that isolation is a shared experience for many ethnic minority women within the perinatal period [30, 31, 32]. Furthermore, current research recommends perinatal teams work across agencies and utilise community resources [9, 31, 33], with further evidence showing that strengthening ethnic minority mothers' support networks and community connections has beneficial effects [34, 35, 36, 37, 38].

Cultural curiosity has emerged as the foundation for the development of cultural knowledge and cultural competency [39]. The study participants identified this as meaningful for building relationships with staff and sharing their identity. This supports Conneely et al.'s [16] exploration of Black and Asian women's experiences with perinatal services, which found that the role of professional cultural curiosity makes women feel heard, accepted and supported. Furthermore, cultural curiosity is essential for professionals to support service users in ‘making sense of how culture, ethnicity, race and racism impact mental health’. This theme was unique compared to other qualitative explorations of ethnic minority women's experiences with perinatal services in that it explicitly identifies the value of professionals and service users making sense of the intersection between mental health difficulties and culture, ethnicity and race. This sense‐making helped women to form a cohesive narrative, experience validation in response to suffering and understand factors contributing towards mental health difficulties.

The final theme, ‘tailoring interventions to ethnic minority women and their families’, is supported by the evidence base for the practical recommendations made by participants, such as linguistic adaptations and flexibility in the length of sessions [40]. A support group for ethnic minority mothers was also recommended, similar to previous studies where ethnic minority participants expressed a need to meet other mothers from similar backgrounds in group settings [31, 32].

### 4.1 Strengths and Limitations

This study has contributed to the evidence base for underresearched and underserved ethnic minority groups accessing perinatal services. Our findings offer valuable insights to both researchers and clinicians for understanding and providing culturally sensitive perinatal care. The group experiential themes present actionable outcomes with clear implications for good practice while preserving the complexity of the phenomena they represent. The clarity of these themes allows for a wider readership, enabling the practical application of the findings [41].

We followed Smith's [42] guide for evaluating IPA research, which outlines the core features of high‐quality IPA studies. For instance, there was a clear focus on the research aims of exploring the experiences of perinatal teams and how cultural sensitivity is conceptualised within this context. Moreover, the study detailed rigour by displaying the prevalence for each theme; for example, Smith [42] advises providing extracts for half the sample in studies with four to eight participants, and Table 3 demonstrates that each theme had extracts from four to six participants. Additionally, the use of conceptual notes facilitated an interpretative analytical approach, with extracts representing the convergence and divergence among participants' perspectives, thereby emphasising the shared yet distinct experiences of ethnic minority women within this sample.

Regarding limitations, the sample size aligns with recommendations for an IPA paper [20]. However, recruitment from perinatal teams was low, and many ethnic minority groups were missing from the sample. Increasing the recruitment window and recruiting from perinatal services across the United Kingdom would help to overcome recruitment challenges and provide a more representative sample. Lastly, the interviews were only conducted in English due to the limited study budget and half of the participants did not speak English as their first language. The results may have been different if the interviews had been conducted in their first language [43].

### 4.2 Clinical Implications

Perinatal teams should prioritise multiagency working, as recommended by the NHS Long‐Term Plan [6], and support women to access peer support and activities within the community. Furthermore, participants expressed how valuable they found discussing their ethnicity and cultural identity with professionals. Naz, Gregory, and Bahu [44] normalise that it is common for White clinicians to lack confidence when talking about culture, ethnicity and race but offer guidance for developing cultural curiosity by discussing culture, ethnicity and race early on in relationships with service users. Clinicians may wish to refer to the Cultural Formulation Interview [45], which encompasses key questions, including, cultural identity, coping, help‐seeking and cultural explanations of illness. Building upon this, clinicians should develop their competencies in supporting service users to make sense of how their culture and mental health influence one another through supportive conversations or psychological formulation.

For interventions, professionals should employ a critically reflective position when using Western models of care. However, clinicians can provide culturally sensitive care across cultures without requiring expertise in numerous models. Our study participants suggested professionals should use appropriate practical, linguistic and cultural adaptations. To expand upon this, clinicians may wish to use the categories of cultural adaptation, recreated by Taylor, Radford, and Calia [46] from Barrera et al. [47], as a guide to determine areas for intervention adaptation where treatment does not inherently incorporate cross‐cultural modifications.

The categories of cultural adaptation include surface‐ and deep‐level components for cultural adaptation. Surface‐level adaptations include the following: (1) linguistic, ensuring that the intervention and associated materials are delivered and translated into a person's preferred language and reading ability, and use of culturally specific language; (2) evidential, integrating narratives and statistics relevant to the cultural group and acknowledging difficult experiences and realities within the group; and (3) peripheral, using activities, images and cultural norms appropriate for a particular group. Deep‐level adaptations include the following: (1) sociocultural, which uses the person's cultural context to formulate and make sense of difficulties in a way that is familiar and understood; (2) constituent‐involving, training and using members of the participant population to enhance engagement [46, 47]. Furthermore, perinatal teams should provide training and resources for staff to develop their culturally sensitive practice [40].

### 4.3 Recommendations for Future Research

Given the differences in perceptions expressed within a culturally diverse sample, further research should focus on participants from specific ethnic minority groups to provide culturally specific recommendations. Furthermore, future research should use interpreters and translation services to include participants who do not speak English to enhance our understanding of culturally sensitive care across a range of cultural and linguistic backgrounds.

## 5 Conclusions

Perinatal services and professionals can provide culturally sensitive and equitable care to ethnic minority women by applying a systemic lens to their recovery; being curious about their culture; supporting them in making sense of how culture, ethnicity, race and mental health interact; and adapting interventions.

## Author Contributions

**Angelene Gardner:** conceptualisation, investigation, writing–original draft, methodology, writing–review and editing, formal analysis, project administration. **Sheri Oduola:** supervision, writing–review and editing, conceptualisation, project administration. **Bonnie Teague:** supervision, writing–review and editing, conceptualisation, project administration.

## Ethics Statement

The NHS Health Research Authority (HRA) granted approval for this research, including ethical approval from Health and Care Research Wales (HCRW), and each participating NHS Trust granted capacity and capability authorisations (REC Reference: 22/SW/0173; IRAS Project ID: 319722).

## Consent

All participants were provided with participant information sheets and were supported in making an informed choice about entering the study. All participants signed consent forms. Participants were informed that they had the right to withdraw and ask for the data's destruction during the data‐gathering phase.

## Conflicts of Interest

The authors declare no conflicts of interest.

## Supporting information

Supporting information.

## Data Availability

Quotes are embedded within the texts, but transcripts will not be openly shared.
